# Exploring the plastome diversity of fifteen centuries-old olive trees (*Oleae europaea* L.) from Jordan: insights and implications for conservation

**DOI:** 10.3389/fpls.2025.1647776

**Published:** 2025-09-09

**Authors:** Nunzio D’Agostino, Ivan Fruggiero, Alessandro Maisto, Francesca Taranto, Mazen A. Al-Kilani, Ayed M. Al-Abdallat

**Affiliations:** ^1^ Department of Agricultural Sciences, University of Naples Federico II, Portici, Italy; ^2^ Institute of Biosciences and Bioresources, National Research Council (CNR-IBBR), Bari, Italy; ^3^ National Center for Agriculture Research (NARC), Amman, Jordan; ^4^ Department of Horticulture and Crop Science, School of Agriculture, The University of Jordan, Amman, Jordan

**Keywords:** DNA barcoding, super-barcode, plastid DNA (cpDNA), genetic diversity, conservation of biodiversity

## Abstract

The olive tree (*Olea europaea* L.) holds exceptional ecological, cultural, and economic significance in the Mediterranean Basin. Understanding its genetic diversity is critical for conservation, breeding, and authentication of olive cultivars. While nuclear genome analyses have elucidated much of the species’ genetic structure, chloroplast genome sequencing provides complementary insights, particularly in tracing maternal lineages, uncovering domestication pathways, and identifying cryptic genetic variation. In this study, we investigated the plastome diversity of fifteen centuries-old olive trees from Jordan through reference-guided assembly and comparative analysis using the FARGA cultivar plastome as a reference. Despite overall genomic conservation, nucleotide diversity analyses revealed several polymorphic hotspots—most notably within the *psbM* and *ycf1* genes and the *atpB-rbcL* intergenic spacer. Structural variation, including simple sequence repeats and tandem repeats, highlighted intra-population diversity. One sample (TF - 3) exhibited heteroplasmy, suggesting a biological origin that warrants further investigation. Phylogenetic reconstruction grouped most samples within the Mediterranean E1 lineage, with TF - 3 and a few others forming distinct clusters. Comparisons with nuclear genotyping data demonstrated both congruence and divergence, emphasizing the value of a dual-genome approach. This study reinforces the utility of plastome sequencing in varietal identification, conservation genetics, and evolutionary studies, and contributes novel genomic resources for Jordanian olive germplasm.

## Introduction

The concept of DNA barcoding involves using a short genetic sequence from a standardized region of the genome to identify and differentiate between species (international barcode of life, https://ibol.org). This method serves as a molecular tool for biodiversity assessment, conservation efforts, and the study of evolutionary relationships. By comparing these sequences among different organisms, researchers can establish distinct genetic signatures, or “barcodes,” that facilitate species identification (Kress and Erickson, 2008).

Early plastid DNA (cpDNA) barcoding primarily relied on a single locus, often resulting in limited resolution and accuracy in species identification. However, advancements in the field have led to the adoption of several key loci recognized for their effectiveness in species identification and phylogenetic studies. The most used loci include *trnH-psbA* intergenic spacer, *maturase K* (*matK*), *ribulose bisphosphate carboxylase/oxygenase large subunit* (*vrbcL*), *psbA-trnH* intergenic spacer, *NADH dehydrogenase subunit F* (*ndhF*), *atpF-atpH* intergenic spacer, *RNA polymerase subunit B* (*rpoB*), and *RNA polymerase subunit B* (*rpoC1*) (Dong et al., 2012). Combining these loci through multi-locus barcoding enhances species identification and phylogenetic analyses by exploiting the unique strengths of each marker (Fazekas et al., 2008; Hollingsworth et al., 2011). Next-generation sequencing (NGS) has further transformed cpDNA barcoding by enabling the analysis of entire plastid genomes, a technique referred to as super-barcoding (Li et al., 2015). This comprehensive genetic framework for species identification addresses the limitations of single- or multi-locus methods, which can sometimes struggle to resolve closely related taxa.

Plastome sequencing is particularly important for the olive tree (*Olea europaea* L.) for several interconnected reasons. First, it enables the detection of genetic variations not easily identifiable through nuclear genomic approaches, offering valuable insights into the phylogenetic relationships among olive subspecies and cultivars. This in-depth genetic understanding supports both conservation and breeding programs, enabling a better assessment of genetic structure and diversity across cultivated and wild olive populations (Besnard et al., 2013; Dong et al., 2022). Such insights shed light on the domestication processes and migration patterns of olive species, especially within the Mediterranean basin, where the olive tree holds substantial ecological and cultural significance (Bazakos et al., 2023; Al-Kilani et al., 2024). Understanding these evolutionary dynamics can guide conservation efforts and ensure the maintenance of valuable genetic resources (Díez et al., 2016; Rallo et al., 2018).

Plastome analysis is valuable for varietal identification, especially in efforts to trace the maternal lineage of cultivars, clonal lines, or wild populations (Besnard et al., 2002a; Mariotti et al., 2023). In *Olea europaea*, accurate varietal identification plays a crucial role in ensuring the genetic authenticity and traceability of olive oil production chains. It safeguards traditional and endemic germplasm and supports the implementation of quality certification schemes, such as Protected Designation of Origin (PDO) and Protected Geographical Indication (PGI) (Pérez-Jiménez et al., 2013; Sion et al., 2021). Recent studies have demonstrated the efficacy of super-barcoding in various plant groups, establishing it as a powerful tool for systematic biology (e.g, Krawczyk et al., 2018; Chen et al., 2023). Additionally, plastome-derived super-barcodes enable precise differentiation among cultivars, thereby enhancing quality control, preventing frauds, and supporting accurate product labeling in the marketplace (Ganopoulos et al., 2013; Kumar et al., 2011). Finally, plastome-based analyses contribute significantly to the conservation of genetic diversity by identifying unique or endangered cultivars, guiding targeted preservation strategies, and reinforcing the long-term resilience of olive agroecosystems.

Early studies, such as Amane et al. (2000), investigated Moroccan olive populations and identified four chlorotypes, emphasizing distinct genetic lineages in wild olives. Building on this, Besnard and Bervillé (2002) compared RFLP and PCR-based methods for cpDNA analysis, demonstrating the superior reliability of PCR-based approaches. Subsequent studies using PCR-RFLP and microsatellite markers identified 15 chlorotypes in the olive complex and constructed a cpDNA phylogenetic tree with five geographically distinct clades: clade A in Central and Southern Africa, clade C in Asia, clade M in North-West Africa, and clades E1 and E2 in the Mediterranean Basin. Cultivated olives clustered with Mediterranean and Saharan wild forms (E1 and E2), showing strong genetic differentiation between eastern and western Mediterranean olives, likely due to distinct glacial refugia. An eastern chlorotype, dominant in cultivars, appears to have been spread westward by humans (Besnard et al., 2002b). Later, Besnard et al. (2011) sequenced eight cpDNA genomes, identifying 22 haplotypes across Mediterranean olive lineages and revealing low nucleotide divergence, particularly in cultivated olives. Mariotti et al. (2010) identified new polymorphic cpDNA regions for improved cultivar differentiation, and Pérez-Jiménez et al. (2013) developed cpDNA markers for varietal tracing in olive oils. In 2015, Noman et al. (2015) advanced the field by creating cpDNA markers for varietal identification, supporting on-farm conservation and preserving authentic olive varieties. Finally, Mariotti et al. (2023) provided deeper insights into domestication by exploring genealogical relationships within olive species.

The goal of this work is to explore the plastome diversity of fifteen centuries-old olive trees from Jordan, providing insights and implications for conservation. These trees were previously genotyped at the nuclear level (Al-Kilani et al., 2024), and here we utilize cpDNA analysis to further elucidate relationships among the samples. Additionally, this study aims to demonstrate the discriminatory power of the complete plastid genome in detecting and identifying mutation hotspots, offering new perspectives for research on biodiversity, phylogeny, and biogeography. Together, these efforts contribute to a deeper understanding of genetic heritage and inform strategies for the preservation of these centuries-old trees.

## Materials and methods

### Plant material, DNA isolation, and sequencing

Leaf samples were collected from fifteen olive trees belong to *Olea europaea* subsp. *europaea*, each estimated to be centuries-old, based on trunk diameter measurements taken at 130 cm above ground level, following the methodology outlined by Arnan et al. (2012), These samples were sourced from various regions across Jordan (see **Table 1**).

**Table 1:** Collection details for the 15 olive trees (Olea *europaea* subsp. *europaea*) investigated in this study, including locations, regions, area names, and climatic zones.

Tree identification code	Location	Region	Area	Latitude	Longitude	Elevation	Physiography	Habitat	Micro-environment
AJ-24	North Jordan	Ajloun	Anjara	32.3060110	35.7525660	877	Plain	Arable	Farmer field
BA-40	Central Jordan	Balaqa	ArRumma	32.1295181	35.6794210	725	Mountain	Forest	Forest margin
BA-42	Central Jordan	Balaqa	ArRumma	32.1295181	35.6794210	725	Mountain	Forest	Forest margin
BA-66	Central Jordan	Balaqa	ArRumma	32.1296770	35.6798990	708	Mountain	Forest	Forest
BA-74	Central Jordan	Balaqa	ArRumma	32.1296310	35.6798770	713	Hill	Forest	Forest margin
BA-75	Central Jordan	Balaqa	ArRumma	32.1296250	35.6798650	709	Hill	Forest	Forest margin
IR-55	North Jordan	Irbid	Kufr-Asad	32.5844280	35.7238330	345	Plain	Arable	Farmer field
IR-61	North Jordan	Irbid	Bani Kana	32.7151360	35.8086270	267	Valley	Rangeland	Rock face
IR-62	North Jordan	Irbid	Bani Kana	32.7151100	35.8086010	267	Valley	Rangeland	Rock face
JA-56	North Jordan	Jarash	Borma	32.2096010	35.7791730	445	Mountain	Forest	Forest
JA-71	North Jordan	Jarash	Borma	32.2158990	35.7991260	470	Mountain	Forest	Forest
JA-72	North Jordan	Jarash	Borma	32.2158860	35.7991220	470	Mountain	Forest	Forest
MN-14	South Jordan	Ma’an	Wadi Musa	29.3358760	35.2861220	1101	Plain	Desert	Sand bank
TF-1	South Jordan	Tafilah	Aema	30.8781990	35.5965200	870	Upland	Arable	Farmer field
TF-3	South Jordan	Tafilah	Aema	30.8781990	35.5965200	870	Upland	Arable	Farmer field

Total genomic DNA (gDNA) was extracted using the Qiagen DNeasy Plant Mini Kit. The integrity of the extracted DNA was assessed through agarose gel electrophoresis, and its concentration was measured using a Qubit^®^ 2.0 fluorimeter (Thermo Scientific).

Approximately 100 ng of gDNA per sample was used as input material for DNA sample preparation. Sequencing libraries were constructed using the NEBNext^®^ Ultra™ DNA Library Prep Kit for Illumina (NEB, USA), in accordance with the manufacturer’s instructions. Index codes were incorporated to distinguish sequences belonging to each sample.

In brief, the DNA samples were fragmented to an average size of 350 bp using sonication. The resulting DNA fragments were then end-polished, A-tailed, and ligated with full-length adapters suitable for Illumina sequencing. Following ligation, PCR amplification was performed to enrich the library. Finally, the PCR products were purified using the AMPure XP system, and the resulting libraries were analyzed for size distribution with an Agilent 2100 Bioanalyzer and quantified via real-time PCR.

### Plastome sequencing, assembling, and annotation

DNA samples were sequenced using the Illumina NovaSeq 6000 platform, generating 2 × 150 paired-end reads. Quality control of the raw reads was performed with FastQC v0.11.9 (https://www.bioinformatics.babraham.ac.uk/projects/fastqc/) and summarized using MultiQC v1.12 (https://seqera.io/multiqc/). Subsequently, Trimmomatic v0.40-rc1 (Bolger et al., 2014) was utilized to eliminate Illumina technical sequences, remove low-quality sequences, trim reads from both ends, filter out any reads that were too short after trimming. The following parameters were applied: adapters.fa:2:20:10:5, LEADING:30, TRAILING:30, SLIDINGWINDOW:10:30, and MINLEN:75.

Reference-based assembly was conducted using NOVOPlasty (Dierckxsens et al., 2016), with the plastome sequence deposited in GenBank under accession LR743800.1 serving as the reference genome. The *matk* gene sequence was used as the seed for the assembly process.

Chloroplast genome annotation was conducted using the GeSeq tool (Tillich et al., 2017), with gene structures manually curated based on the structural annotations of reference sequence LR743800.1.

The circular plastome map of sample AJ - 24 was generated using the online tool OGDRAW (Greiner et al., 2019).

Chloroplast genome sequences and annotations generated in this study are available in GenBank under accession numbers PQ676988-PQ677002.

### Identification and characterization of sequence variations

Nucleotide variability among olive cpDNAs was analyzed utilizing a variety of bioinformatic tools.

To enable comparative analysis, multiple sequence alignment was performed using the MAFFT program (Katoh and Standley, 2013). The analysis included the plastome of *Olea europaea* subsp. *europaea* cultivar FARGA (accession LR743800.1) along with the following reference genomes deposited in GenBank: NC_015401.1 (*Olea europaea* var. *sylvestris*), MW072292.1 (*Olea europaea* subsp. *europaea* cultivar Mehras), and CM032646.1 (*Fraxinus excelsior*).

RAxML (Stamatakis, 2014) was employed to construct a maximum-likelihood (ML) tree with 1,000 rapid bootstrap inferences, using a generalized time reversible (GTR) substitution model and the Gamma model of rate heterogeneity. In parallel, a neighbor-joining (NJ) tree was generated in MEGA 11 (Tamura et al., 2021; https://www.megasoftware.net) with 1,000 bootstrap replicates. The plastome of *F. excelsior* was designated as the outgroup for both analyses. Tree visualizations were performed using FigTree v1.4.2 (http://tree.bio.ed.ac.uk/software/figtree/).

The identification of microsatellites was performed using the MISA web tool (Beier et al., 2017), available at https://webblast.ipk-gatersleben.de/misa/. The unit size and minimum repeat parameters were set as follows: 1/10, 2/6, 3/5, 4/5, 5/5, and 6/5. Additionally, tandem repeats were detected using the Tandem Repeat Finder (TRF) web tool, accessible at https://tandem.bu.edu/trf/home, with default settings applied (Benson, 1999).

Single nucleotide variants (SNVs) were extracted from a multiple sequence alignment using the *snp-sites* tool (Page et al., 2016), with the plastome LR743800.1 as the reference sequence. A custom Python script was then developed to determine whether the SNVs were located within genic or intergenic regions.

Nucleotide diversity (π) was assessed using a sliding window approach. The π calculations were performed iteratively with the MSA2pi tool (https://github.com/baoxingsong/MSA2pi), which lacks support for overlapping window configurations. To overcome this limitation, a custom Python script was developed to segment the multiple sequence alignment and automate the execution of MSA2pi. Additionally, a second Python script was created to include both single nucleotide polymorphisms (SNPs) and insertions/deletions (indels) in the diversity calculation. The analysis utilized a window size of 1000 base pairs (bp) and a step size of 500 bp. The results were stored in a comma-separated values (CSV) file for subsequent analysis. Graphical representations of the nucleotide diversity data were produced using Python, with the *pandas* and *matplotlib.pyplot* libraries for visualization.

## Results

### Plastome size and organization

A reference-guided assembly was conducted using the cpDNA of the FARGA cultivar (LR743800.1) as the reference to obtain the complete plastome for all 15 samples. Ten samples exhibited an identical genome size of 155,886 bp, while the remaining five showed variations of one or two base pairs, either larger or smaller (**Table 2**). Notably, the sequence of sample MN - 14 is identical to that of JA - 72 and IR - 62. As expected, all genotypes exhibited the characteristic quadripartite structure typical of angiosperms, consisting of a pair of inverted repeats (IR) of 25,741 bp, flanked by two single-copy regions: a large single copy (LSC) ranging from 86,610 bp to 86,614 bp, and a small single copy (SSC) of 17,791 bp (**Table 2**). No variation in guanine/cytosine (GC) content was observed among the genotypes, with all showing a consistent GC content of 37%.

**Table 2:** Plastome features of the olive tree samples sequenced in this study.

Tree identification code	GenBank acc.	Size (base pairs)			
		Total	LSC	SSC	IR
AJ-24	PQ676988	155887	86612	17791	25742
BA-40	PQ676989	155885	86610	17791	25742
BA-42	PQ676990	155886	86611	17791	25742
BA-66	PQ676991	155886	86611	17791	25742
BA-74	PQ676992	155886	86611	17791	25742
BA-75	PQ676993	155886	86611	17791	25742
IR-55	PQ676994	155888	86613	17791	25742
IR-61	PQ676995	155886	86611	17791	25742
IR-62	PQ676996	155886	86611	17791	25742
JA-56	PQ676997	155885	86610	17791	25742
JA-71	PQ676998	155886	86611	17791	25742
JA-72	PQ676999	155886	86611	17791	25742
MN-14	PQ677000	155886	86611	17791	25742
TF-1	PQ677001	155886	86611	17791	25742
TF-3	PQ677002	155884	86609	17791	25742

The plastid DNA contains 125 unique, non-redundant genes, including 80 protein-coding genes, 30 *tRNA* genes, and 4 *rRNA* genes. Of the *tRNA* genes, 7 are located within the inverted repeat (IR) regions and are duplicated, as are the *rRNA* genes. The LSC region contains 60 protein-coding genes, 7 of which are multi-exon, and 22 *tRNA* genes, 4 of which have two exons. The SSC region includes 11 protein-coding genes, one with two exons, and a single *tRNA* gene.

**Figure 1:**
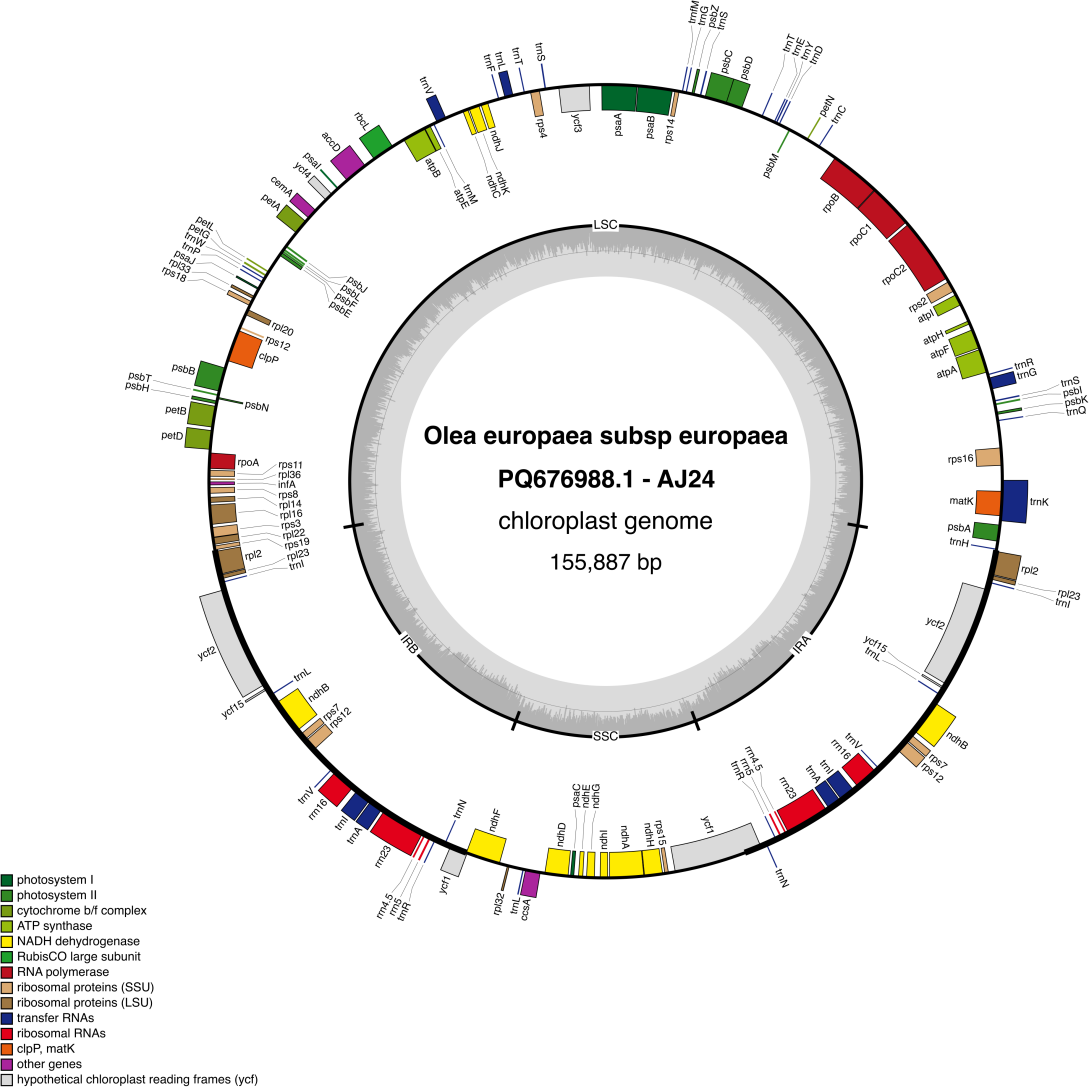
Map of the AJ - 24 chloroplast genome. Genes located inside the outer circle are transcribed in the clockwise direction, while those outsides are transcribed counterclockwise. Different colors represent genes classified into various functional groups. The inner circle highlights the GC content, with the 50% threshold marked for reference. The genome is divided into the inverted repeat (IR), large single-copy (LSC), and small single-copy (SSC) regions, as indicated by their respective labels.

The IR regions feature 8 protein-coding genes, including a truncated copy of *ycf1*, 7 *tRNA* genes (2 with two exons), and the 4 *rRNA* genes. The *rps12* gene is trans-spliced, with exons 2 and 3 in the IR. Additionally, *rps19* spans the LSC–IRb junction (**Figure 1**).

### Nucleotide variability and phylogenetic insights

**Figure 2:**
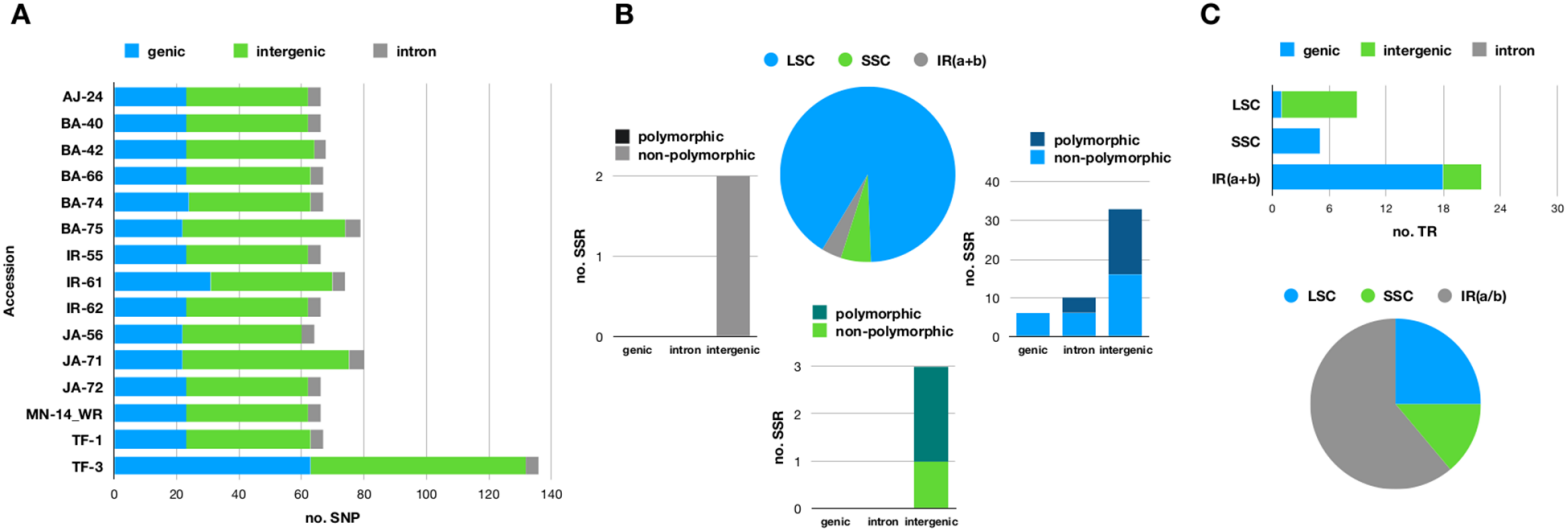
**(A)** Stacked bar chart illustrating the distribution of single nucleotide polymorphisms (SNPs) across genic, intronic, and intergenic regions in the fifteen olive plastomes sequenced in this study. **(B)** Pie chart illustrating the percentage of microsatellites (SSRs) located in the large single-copy (LSC), small single-copy (SSC), and inverted repeat (IR) regions. Each slice of the pie corresponds to a stacked bar chart that categorizes microsatellites into genic, intronic, and intergenic regions. The bars are further divided into different shades to indicate whether the microsatellites are polymorphic or not. **(C)** Pie chart depicting the percentage of tandem repeats (TRs) found in the LSC, SSC, and IR regions. Each slice of the pie is linked to a stacked bar chart that further classifies microsatellites into genic, intronic, and intergenic regions. The plastome of the FARGA cultivar (LR743800.1) served as the reference for investigating nucleotide variability.

**Figure 2A** provides an overview of nucleotide variability. Of the 158 variable loci, 89 were located in intergenic regions, while the remaining 69 were found within 23 genes (see **Supplementary Table 1**). SNP counts varied across samples, ranging from 64 in JA - 56 to 136 in TF - 3 (**Figure 2A**). The large number of SNPs observed in the TF3 sample is attributed to loci in a heterozygous state. We investigated whether these loci are the result of sequencing errors or due to heteroplasmy. As shown in **Supplementary Figure 1**, a large percentage of sequence reads confirm the presence of the alternative allele, supporting the hypothesis that heteroplasmy is the underlying cause.

Fifty-four simple sequence repeats (SSRs) were identified, including 4 compound repeats (**Figure 2B**). Fourteen of these microsatellites were located within 8 genes, with *rpoC2* containing 4 SSRs, *clpP* having 3 SSRs, and *rpl16* containing 2 SSRs. Mononucleotide repeats (adenosine or thymine) were the most common type of microsatellite. Twenty-three loci exhibited variable numbers of repetitive units (**Figure 2B**; **Supplementary Table 2**).

A total of 25 tandem repeats were identified, with 11 located in the IR regions (**Figure 2C**). Their sizes ranged from 9 to 36 bases, and repeat periods varied between 2 and 7.4 bases. Among these, 10 tandem repeats were found in intergenic regions, while the remaining 15 were associated with four genes (**Figure 2C**), with nine specifically localized within the *ycf2* gene.

Notably, a tandem repeat with a period size of 12 bases and a copy number of 3.2 was absent in the reference genome but present in all sequenced samples. Conversely, a unique tandem repeat with a period size of 18 bases and a copy number of 2 was found exclusively in the reference sequence LR743800.1 (**Supplementary Table 3**).

**Figure 3:**
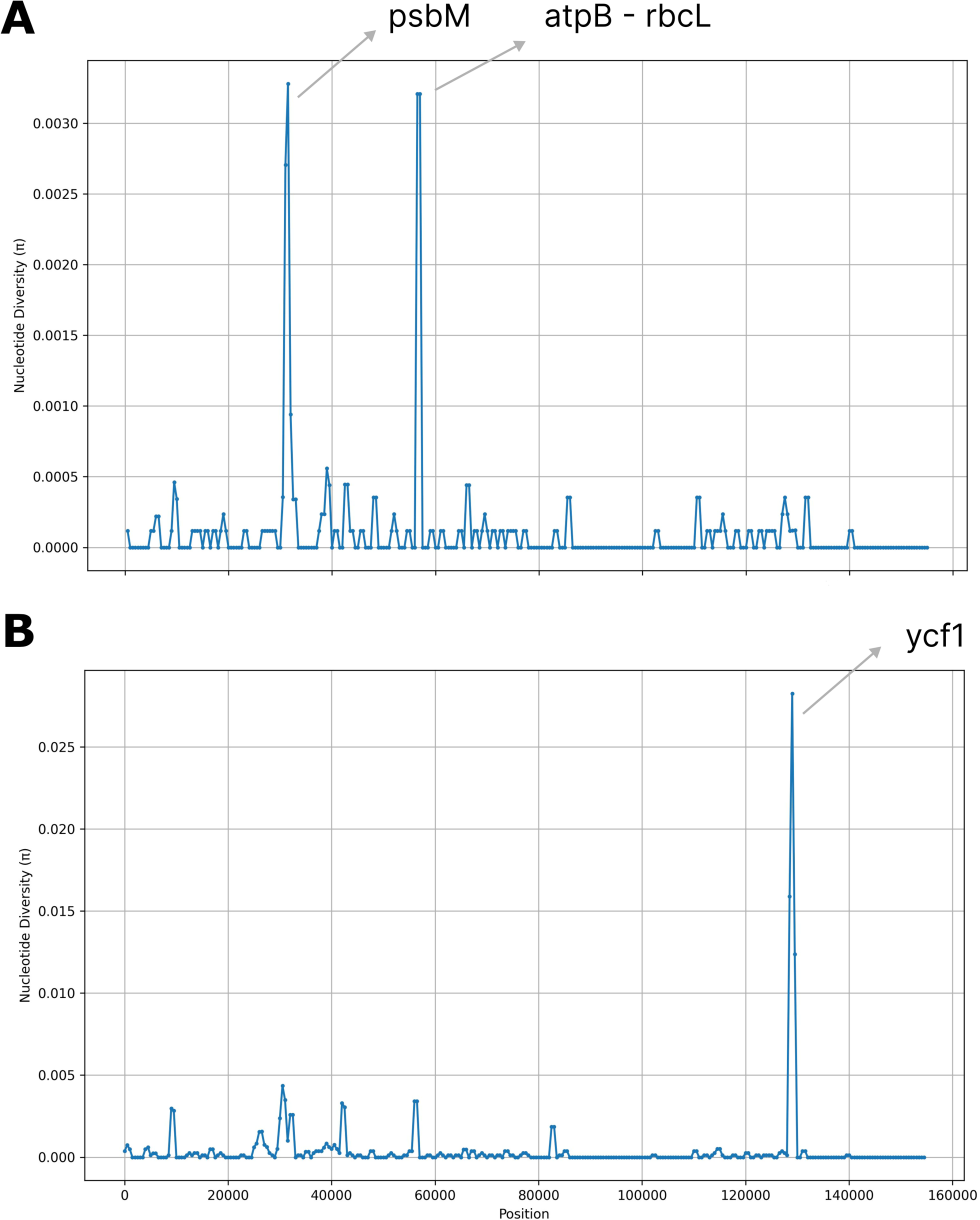
Nucleotide diversity (π) values across 17 complete olive plastomes. Regions with high nucleotide variability (π > 0.025) are highlighted. **(A)** Single nucleotide polymorphisms (SNPs). **(B)** Insertions and deletions (indels). Window size = 1000 base pairs (bp); step size = 500 bp.

The nucleotide diversity (π) analysis revealed a high degree of sequence similarity across the olive plastomes while identifying three main polymorphic hotspots: two within genic regions (*psbM* and *ycf1*) and one in the intergenic *atpB-rbcL* spacer. Notably, *psbM* and *atpB-rbcL* exhibited high SNP variability, whereas *ycf1* showed a higher frequency of indels (**Figure 3**). Specifically, the ycf1 protein in the reference plastome is 1,801 amino acids long, whereas in all analyzed samples, it extends to 1,876 amino acids. The pairwise global alignment highlights a 75-amino acid insertion in the ycf1 protein of AJ - 24. A screenshot of this alignment, comparing the ycf1 protein in the reference sequence (LR743800.1) and AJ - 24, is provided in **Supplementary Figure 2**.

**Figure 4:**
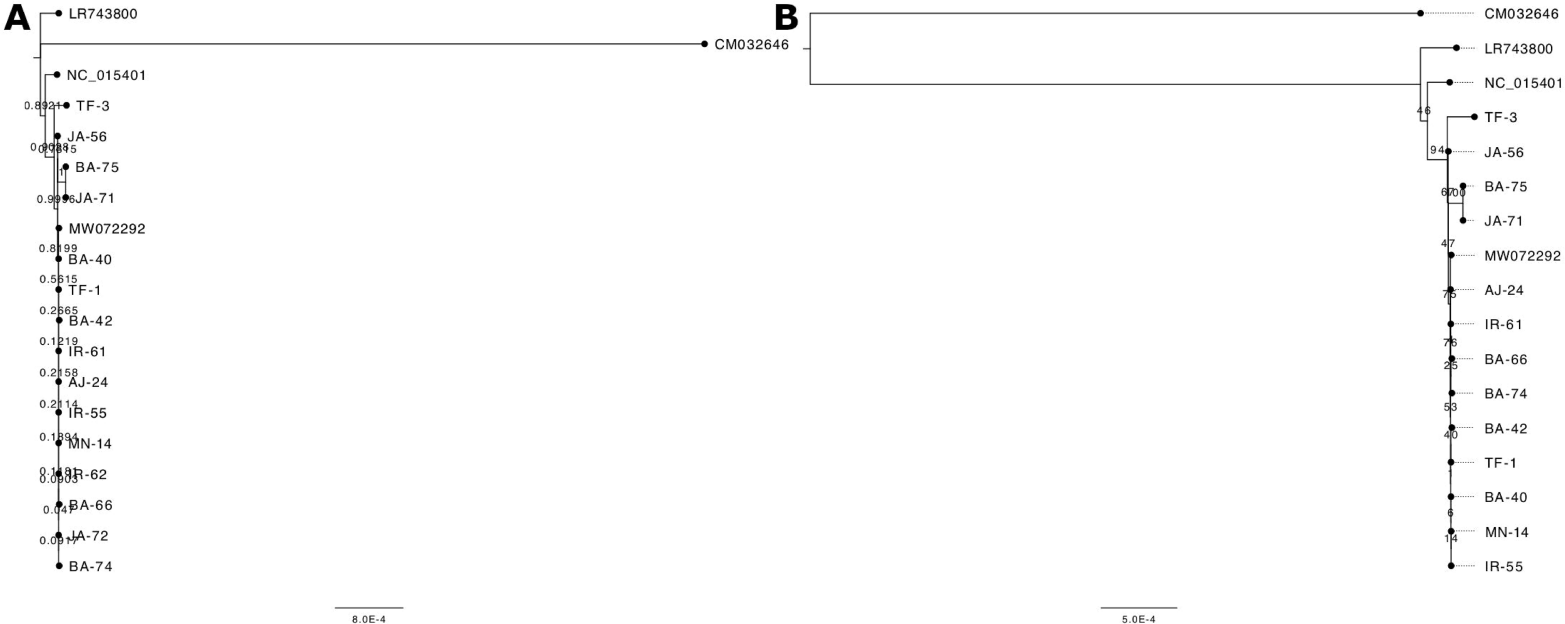
Phylogenetic trees constructed using **(A)** the Neighbor-Joining method and **(B)** the Maximum Likelihood method. The analysis included 15 Jordanian olive samples alongside reference sequences: LR743800.1 (Olea europaea subsp. europaea), NC_015401.1 (Olea europaea var. sylvestris), MW072292.1 (Olea europaea subsp. europaea, cultivar MEHRAS), and CM032646.1 (Fraxinus excelsior), with F. excelsior serving as the outgroup. Bootstrap support values are indicated on the branches. The tree in panel **(B)** contains two fewer samples, as their sequences were identical to MN - 14 and were removed from the analysis.

Two distinct algorithms were utilized to investigate the phylogenetic relationships among the samples under study. **Figure 4** illustrates the phylogenetic trees generated using the Maximum Likelihood (ML) algorithm (**Figure 4A**) and the Neighbor Joining (NJ) method (**Figure 4B**). These trees provide a visual representation of the evolutionary relationships among the analyzed samples, highlighting both genetic similarities and differences. Bootstrap values placed near the nodes indicate the level of statistical support for each grouping, with higher values suggesting stronger confidence in the tree’s structure. In the ML-based phylogenetic tree (**Figure 4A**), a distinct cluster is formed by samples JA - 56, BA - 75, JA - 71, and TF - 3, which group together with a bootstrap value of 100, signifying a high degree of genetic similarity among them. The NJ-based phylogenetic tree (**Figure 4B**) shows that the majority of the Jordanian samples are positioned in the upper portion of the tree, implying a closer evolutionary relationship between them. The reference sequences, LR743800.1 and NC_015401.1, are found in the lower section, confirming their role as benchmark sequences for comparison against the analyzed samples. CM032646.1 is designated as an outgroup and is located at the base of both trees. This outgroup serves to root the tree and provides a reference point for determining the evolutionary direction.

## Discussion

### Plastome features and their barcoding potential

Using complete plastome sequences as DNA barcodes offers a groundbreaking method for identifying and characterizing *Olea europaea* species and cultivars. Traditional barcoding, based on short genetic markers, often fails to distinguish closely related taxa. Advances in next-generation sequencing now allow for full plastid genome analysis, improving resolution in complex cases (Krawczyk et al., 2018; Song et al., 2020; Li et al., 2015; Coissac et al., 2016).

Olive plastomes, which exhibit relatively low mutation rates and highly conserved structures, provide a robust framework for species identification and phylogenetic studies. The use of plastomes as barcodes leverages the unique advantages of these genomic regions, allowing for the identification of distinct genetic signatures associated with different olive subspecies and cultivars. This enhanced resolution is particularly beneficial in regions with rich biodiversity, such as the Mediterranean basin, where olives have been cultivated for millennia (Besnard et al., 2018).

This method can detect cryptic diversity among phenotypically similar cultivars, aiding in taxonomy, conservation, and the understanding of evolutionary relationships (Gostel and Kress, 2022; Kartzinel et al., 2025).

Challenges include the need for comprehensive plastome databases and standardized protocols to ensure reproducibility across studies (Freudenthal et al., 2020; Turudić et al., 2021; Heinze, 2021).

Moreover, plastid diversity in cultivated olives is limited, with strong plastome-mitochondrial linkage indicating minimal recurrent organellar mutations (Mariotti et al., 2010).

### Genomic variation and polymorphic hotspots

SNP identification reveals a higher degree of variability in non-coding regions compared to coding regions. The number of SNPs varied across samples (**Supplementary Table 1**), which may reflect population-specific variations or the influence of environmental factors.

Notably, the TF - 3 sample exhibits heteroplasmy at multiple loci, indicating the possible presence of different plastid genome variants within the same organism. Plastid heteroplasmy, defined as the coexistence of multiple sequence variants in a single cell, challenges the conventional view of plastomes as predominantly homoplasmic due to their clonal inheritance and high copy number. The occurrence of heteroplasmy at different loci within the chloroplast genome suggests the involvement of underlying biological mechanisms that warrant further investigation. Several factors may contribute to this heteroplasmic state, including biparental inheritance, somatic mutations, and intracellular recombination (Greiner et al., 2015). In the TF - 3 sample, the presence of distinct alleles has been independently confirmed by consistent read coverage, allowing us to rule out sequencing artifacts or assembly errors. Therefore, further studies are needed to elucidate the biological basis of chloroplast heteroplasmy in this case.

Twenty-three microsatellite loci exhibited variable numbers of repetitive units (**Supplementary Table 2**), which could be crucial for understanding the genetic diversity and stability of these loci across different samples. Notably, a tandem repeat with a size of 18 bases and a copy number of 4.2 was absent in samples BA - 40 and BA - 42, suggesting this repeat may be polymorphic or that these samples have undergone genomic variation leading to its loss. In contrast, a tandem repeat with a period size of 12 bases and a copy number of 3.2 was absent in the reference genome but present in all sequenced samples, highlighting its potential significance or uniqueness in the sequenced populations. Furthermore, a unique tandem repeat with a period size of 18 bases and a copy number of 2 was found exclusively in the reference sequence LR743800.1 (**Supplementary Table 3**), indicating that this repeat may be specific to the reference genome and could serve as a valuable marker for further comparisons. The nucleotide diversity (π) analysis identified three polymorphic hotspots. The *psbM* gene, encoding a small subunit of photosystem II, is not typically reported as a major hotspot for plastome polymorphism. However, genes involved in photosynthesis can accumulate variations due to environmental selection pressures or lineage-specific mutations.

The high SNP variability observed in *psbM* among olive plastomes could potentially result in structural variations of the PSBM protein, which might influence its function within the photosystem II complex. Assessing the amino acid substitutions and their locations relative to critical functional domains or interaction sites could provide insight into the possible structural and functional impacts of these variations.

The *atpB-rbcL* intergenic spacer is widely recognized as a polymorphic hotspot in plastomes across various plant species. Intergenic spacers, particularly those between essential photosynthetic genes like *atpB* and *rbcL*, tend to accumulate mutations more readily due to relaxed selective constraints. They are often used as molecular markers in phylogenetic and population genetics studies due to their high variability (Shaw et al., 2007). Finally, the *ycf1* gene is one of the most well-documented polymorphic regions in plastomes. It plays a role in protein translocation and is known for its high evolutionary rate. Studies across diverse angiosperms have consistently identified *ycf1* as one of the most variable plastid genes, making it a valuable marker for phylogenetic and evolutionary studies (Dong et al., 2015). The observed 75-amino acid insertion in AJ - 24 and other Jordanian samples suggests a structural modification, which may have functional or evolutionary significance.

### Phylogenetic clustering and lineage differentiation

The phylogenetic analysis revealed distinct clustering patterns among the 15 selected olive trees from Jordan, with the NJ tree including all samples while the ML tree represented 13. Notably, samples IR - 62 and JA - 72 were absent from the ML tree (as they were identical to MN - 14), yet they appeared identical in the NJ analysis. The clustering of the majority of Jordanian olive trees within the E1 lineage, which includes the Mehras cultivar (referred to as Kfari Baladi by Al-Kilani et al., 2024), indicates a significant genetic affinity among these trees. Importantly, no samples were found to cluster with Haut Atlas (E2 lineage) or Farga (E3 lineage), suggesting a distinct genetic landscape for the olive trees in Jordan.

Among the analyzed samples, TF - 3 stands out for its marked divergence from the other Jordanian samples. This distinction is particularly intriguing given TF - 3’s location in southern Jordan, a region geographically isolated from the northern areas. This divergence warrants further investigation, as it could offer valuable insights into localized adaptations or historical events that contributed to genetic separation. Interestingly, TF - 3 formed a distinct cluster with JA - 56, BA - 75, and JA - 71 in both phylogenetic trees, clearly separating them from the remaining samples. In contrast, nuclear genotyping of the same samples in a previous study (Al-Kilani et al., 2024) placed them within a larger cluster that encompassed the majority of genetically diverse trees from Jordan.

Interestingly, the predominant cultivars Kfari Baladi (Mehras), Kanabsi (MN - 14, Wadi Rum), and BA - 66 were found to be genetically identical, aligning with previous studies indicating their classification within the E1 lineage. This observation highlights the need for nuclear-level genetic analysis to further differentiate these cultivars, as plastid data alone may not capture the full extent of their genetic diversity.

These findings are consistent with the broader history of olive domestication, which began in the Eastern Mediterranean—particularly the Levant, including present-day Jordan—before spreading westward through trade and migration (Besnard et al., 2013). Eastern olive germplasm played a key role in shaping cultivated varieties across the Mediterranean, as reflected in the widespread E1 plastid lineage (Julca et al., 2020). Secondary domestication events through admixture with local wild populations further contributed to modern olive diversity.

Recent genomic studies underscore this complexity, revealing recurrent admixture (Julca et al., 2020), crop-to-wild gene flow in the west (Zunino et al., 2023), and genetic links across Mediterranean lineages (Bazakos et al., 2023; Mariotti et al., 2023), supporting a multi-regional domestication model.

However, data on historical gene flow involving Jordanian germplasm remain limited. This highlights the need for expanded nuclear and plastome analyses and regional phylogeographic studies to clarify Jordan’s role in Mediterranean olive diversity. Future work will integrate plastid, nuclear, and morphological data to address this gap.

### Integrating nuclear and plastome genotyping for evolutionary, agricultural, and conservation insights

All samples analyzed in this study were previously subjected to nuclear genotyping (Al-Kilani et al., 2024). Nuclear genotyping successfully separated Jordanian centuries-old olive trees into distinct sub-groups and identified 73 previously unreported olive genotypes, 15 of which were used in this study. In contrast, plastome genotyping grouped together some genotypes that had been separated by nuclear markers, while also revealing clear variation among others. The integration of both nuclear and plastid genomic data enables researchers to capitalize on their complementary inheritance patterns, mutation rates, and functional roles, providing a more comprehensive framework for investigating evolutionary relationships, population structure, and adaptive traits. By combining these two genomic approaches, this study not only contributes valuable genomic resources but also demonstrates the power of integrated genotyping to deepen our understanding of plant genetics and evolutionary dynamics.

One of the key findings that underscores this integrative approach is the enhanced phylogenetic resolution achieved by combining nuclear and plastome genotyping. Each genome offers distinct insights into evolutionary history: nuclear DNA, inherited biparentally and subject to recombination, reflects recent divergence and gene flow, while plastome DNA, with its uniparental inheritance and lower mutation rate, is better suited for tracing deep evolutionary lineages and maternal ancestry. This dual-genome approach thus enables the reconstruction of both recent and ancient evolutionary pathways, which could otherwise remain ambiguous if either genome were studied in isolation.

Furthermore, this study underscores the utility of combined genotyping in elucidating cytoplasmic-nuclear interactions, which play a significant role in plant fitness and adaptability. Cytoplasmic genomes, such as those of chloroplasts, encode essential components of photosynthesis and other metabolic pathways, often interacting with nuclear-encoded factors to produce key adaptive traits. For instance, environmental stress responses and energy metabolism are often the product of complex interactions between nuclear and chloroplast genes. By analyzing both genomes simultaneously, researchers can better understand these interactions and identify specific nuclear-plastid genotype combinations that contribute to environmental resilience. This insight has practical applications in agriculture, particularly in the development of crops with enhanced tolerance to abiotic stresses such as drought or high temperatures (Zhang et al., 2020).

Additionally, the combined approach offers significant advantages for studying population structure and gene flow in plant species.

The evolutionary history and domestication of the olive have been extensively studied using both nuclear and plastid DNA markers. Plastome genotyping, which follows maternal inheritance, has been instrumental in identifying distinct chloroplast lineages and tracing seed dispersal patterns across the Mediterranean Basin. For example, two main oleaster (wild Mediterranean olive) genepools have been detected in the Western and Eastern Mediterranean, with specific plastid lineages showing geographic differentiation (Besnard et al., 2018).

In contrast, nuclear genotyping, reflecting biparental inheritance, reveals pollen-mediated gene flow and tends to show weaker geographic structure due to recurrent gene flow and metapopulation dynamics influenced by environmental changes. This complementary use of plastid and nuclear markers helps clarify olive biogeography, domestication, and diversification processes (Besnard et al., 2018).

The complementary insights gained from nuclear and plastome marker systems deepen our understanding of olive biogeography, domestication, and diversification. By distinguishing between pollen- and seed-mediated gene flow, researchers can better assess genetic connectivity within populations—a critical factor for effective conservation strategies, particularly in fragmented habitats where gene flow may be limited. Maternal lineages tracing via plastome genotyping further supports the identification and preservation of distinct genetic lineages, helping preserve the adaptive potential of vulnerable olive populations.

Finally, our study demonstrates the broader value of combined nuclear and plastome data for conservation genetics and biodiversity studies. This dual-genome approach ensures the preservation of genetic diversity across both biparental and uniparental lineages, which is essential for maintaining a species’ adaptive potential and resilience in the face of environmental changes.

While the limited sample size (n=15) poses constraints on the generalizability of our findings, it reflects the realities of working with centuries-old, protected trees and logistical limitations. Nonetheless, these samples were strategically selected to represent key genetic clades identified in previous research (Al-Kilani et al., 2024), ensuring meaningful insight into the Jordanian olive genetic pool. We are actively working to conserve Jordan’s ancient olive germplasm through the establishment of a national gene bank. This initiative will support broader sampling and more comprehensive genetic studies, enabling deeper exploration of gene flow and lineage connections between Jordan and other Mediterranean regions.

## Conclusion

Overall, plastome sequencing offers a comprehensive approach to enhancing our understanding of olive tree genetics, supporting conservation efforts, and contributing to the agricultural and economic sustainability of olive cultivation.

In conclusion, the application of olive plastomes as barcodes represents a significant advancement in the fields of botany, conservation, and agriculture. By harnessing the power of complete plastid genome sequencing, researchers can enhance species identification, uncover genetic diversity, and support conservation and breeding initiatives. The integration of plastome barcoding into olive research has the potential to transform our understanding of olive biodiversity and strengthen the sustainability of olive cultivation in a rapidly changing world.

## Data Availability

The datasets presented in this study can be found in online repositories. The names of the repository/repositories and accession number(s) can be found below: https://www.ncbi.nlm.nih.gov/genbank/, PQ676988–PQ677002 https://www.ncbi.nlm.nih.gov/sra/, PRJNA1182319.

## Supplementary material

Supplementary Table 1 Single-nucleotide polymorphisms (SNPs) identified in the 15 olive chloroplast genomes analyzed, using FARGA (LR743800.1) as the reference plastome. The figure presents SNP alleles per sample and their distribution across genic and intergenic regions of the plastome.

Supplementary Table 2 Simple sequence repeats (SSRs) identified in the 15 olive chloroplast genomes analyzed, using FARGA (LR743800.1) as the reference plastome. The table presents SSR size, location, and distribution across genic and intergenic regions of the plastome.

Supplementary Table 3 Tandem repeats (TRs) identified in the 15 tomato chloroplast genomes using FARGA (LR743800.1) as the reference plastome. The table presents TR copy number, genomic location, and TR distribution across genic and intergenic regions of the plastome.

Supplementary Figure 1 100% stacked bar chart illustrating the allele composition at variable sites in the TF - 3 sample. Each bar represents a distinct variable site, with segments indicating the proportion (%) of reference and alternate alleles.

Supplementary Figure 2 A screenshot of the protein alignment comparing the ycf1 gene product in the reference sequence (LR743800.1) and the AJ - 24 sample. The pairwise global alignment reveals a distinct 75-amino acid insertion in the ycf1 protein of the AJ - 24 sample, which is absent from the reference sequence, indicating a potential structural divergence.
