# Supplementary figures and images for: Exploring the plastome diversity of fifteen centuries-old olive trees (Oleae europaea L.) from Jordan: insights and implications for conservation
Source: Front Plant Sci. 2025 Sep 9;16:1647776. doi: 10.3389/fpls.2025.1647776 (PMC12454372; doi:10.3389/fpls.2025.1647776)

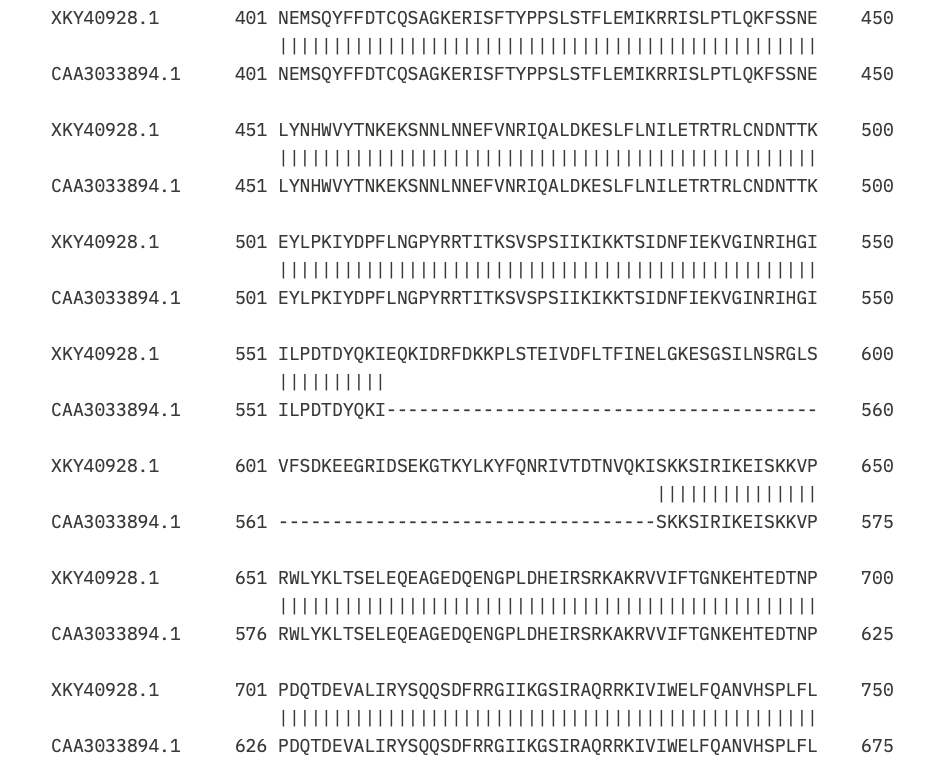

Supplement: Supplementary Figure 1 — 100% stacked bar chart illustrating the allele composition at variable sites in the TF - 3 sample. Each bar represents a distinct variable site, with segments indicating the proportion (%) of reference and alternate alleles. [file Image1.tiff]

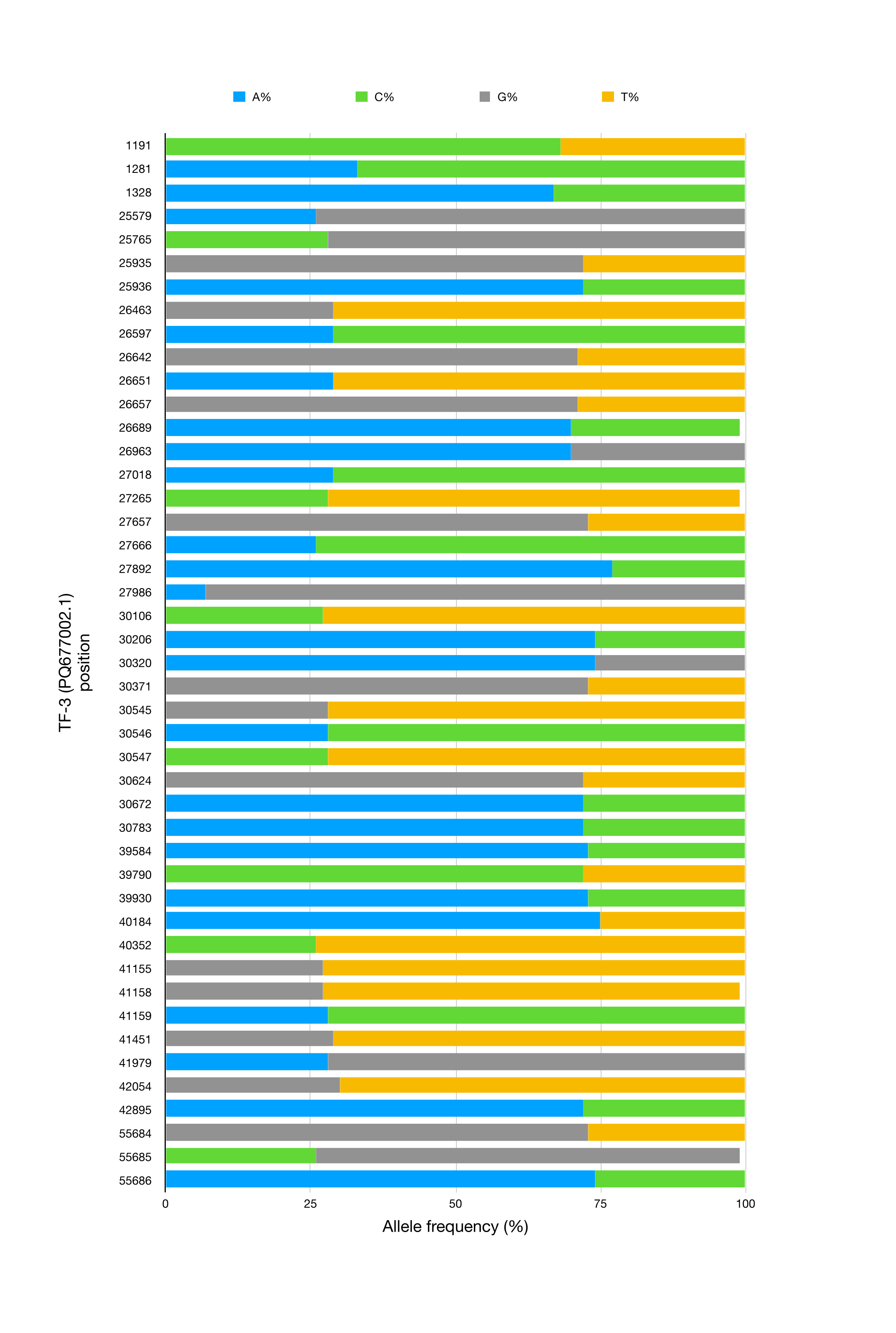

Supplement: Supplementary Figure 2 — A screenshot of the protein alignment comparing the ycf1 gene product in the reference sequence (LR743800.1) and the AJ - 24 sample. The pairwise global alignment reveals a distinct 75-amino acid insertion in the ycf1 protein of the AJ - 24 sample, which is absent from the reference sequence, indicating a potential structural divergence. [file Image2.tiff]
